# ^1^H‐NMR screening for the high‐throughput determination of genotype and environmental effects on the content of asparagine in wheat grain

**DOI:** 10.1111/pbi.12364

**Published:** 2015-03-27

**Authors:** Delia I. Corol, Catherine Ravel, Marianna Rakszegi, Gilles Charmet, Zoltan Bedo, Michael H. Beale, Peter R. Shewry, Jane L. Ward

**Affiliations:** ^1^ Department of Plant Biology and Crop ScienceRothamsted ResearchHarpendenHertfordshireUK; ^2^ INRA‐UBPUMR1095 GDECClermont‐Ferrand CedexFrance; ^3^ Agricultural InstituteCentre for Agricultural Research of the Hungarian Academy of SciencesMartonvásárHungary

**Keywords:** Asparagine, ^1^H‐NMR, wheat, metabolite profiling, G × E, heritability

## Abstract

Free asparagine in cereals is known to be the precursor of acrylamide, a neurotoxic and carcinogenic product formed during cooking processes. Thus, the development of crops with lower asparagine is of considerable interest to growers and the food industry. In this study, we describe the development and application of a rapid ^1^H‐NMR‐based analysis of cereal flour, that is, suitable for quantifying asparagine levels, and hence acrylamide‐forming potential, across large numbers of samples. The screen was applied to flour samples from 150 bread wheats grown at a single site in 2005, providing the largest sample set to date. Additionally, screening of 26 selected cultivars grown for two further years in the same location and in three additional European locations in the third year (2007) provided six widely different environments to allow estimation of the environmental (E) and G x E effects on asparagine levels. Asparagine concentrations in the 150 genotypes ranged from 0.32 to 1.56 mg/g dry matter in wholemeal wheat flours. Asparagine levels were correlated with plant height and therefore, due to recent breeding activities to produce semi‐dwarf varieties, a negative relationship with the year of registration of the cultivar was also observed. The multisite study indicated that only 13% of the observed variation in asparagine levels was heritable, whilst the environmental contribution was 36% and the GxE component was 43%. Thus, compared to some other phenotypic traits, breeding for low asparagine wheats presents a difficult challenge.

## Introduction

Free amino acids generally account for about 5% or less of the total nitrogen content of wheat grain, with asparagine accounting for 10% or less of the total (reviewed by Lea *et al*., 2007). Consequently this amino acid was of little scientific interest or practical importance until the demonstration that it is a precursor of acrylamide which is formed in processed foods by a Maillard reaction with reducing sugars (Mottram *et al*., 2002; Stadler *et al*., 2002). Thus, acrylamide, which has neurotoxic and carcinogenic properties, may be present in cooked foods at concentrations up to 1 mg/kg (Friedman, 2003; Tareke *et al*., 2002). Furthermore, the formation of acrylamide is correlated with the free asparagine content of wheat flour, rather than the content of reducing sugars (Muttucumaru *et al*., 2006, 2008). Consequently the control of asparagine synthesis and accumulation is of considerable current interest to food processors and in contemporary crop science. The development of high‐throughput analytical screens to monitor asparagine levels in crops and crop products is essential to support breeding and selection programmes aimed at reducing the risk of acrylamide formation.

Most recent studies have utilized GC, GC‐MS or LC‐MS of partially purified, and derivitised, amino acid fractions, as analytical techniques for asparagine quantitation. An early study indicated that asparagine accumulated in cereal grain under conditions of sulphur deficiency (Shewry *et al*., 1983) and this has been confirmed by more recent studies (Muttucumaru *et al*., 2006). The asparagine content of wheat grain also increases with protein content (Claus *et al*., 2008) raising particular concerns for the production of bread‐making wheats. However, substantial variation in the asparagine content of wheat grain has been reported which does not appear to be related to nutritional status alone (Baker *et al*., 2006; Claus *et al*., 2006), indicating the impact of other environmental factors. Furthermore, although variation has been reported in the contents of asparagine in different wheat cultivars grown at six locations in the UK (Curtis *et al*., 2009), the relative effects of genotype and environment, and possible interactions between these, have not been quantified in large scale studies across diverse locations and environments. However, analysis of five rye cultivars grown in six European environments showed that only 23% of the total variance in free asparagine concentration was attributed to the effect of variety (Curtis *et al*., 2010). More recently, a detailed comparison was reported of 92 wheat varieties grown in two glasshouse experiments. This showed that the broad sense heritability for asparagine was low (32%) but nevertheless identified possible SNP markers for breeding (Emebiri, 2014).

The rye samples analysed by Curtis *et al*. (2010) were provided by the EU FP6 HEALTHGRAIN programme which was focussed on providing health benefits to consumers by increasing the consumption of protective compounds in whole grains or their fractions (Poutanen *et al*., 2008). This study included a detailed analysis of genetic diversity in the composition of wheat grain, with 150 bread wheat lines and 50 lines of other cereals (including the ‘ancient’ wheats einkorn, emmer and spelt) being initially grown on a single site in Hungary in 2005 (Ward *et al*., 2008). A smaller set of 26 lines, including 23 from the initial screen, were then grown on the same site for two further years (2006, 2007) and on three further sites (in France, UK and Poland) in 2007 only (Shewry *et al*., 2010). This material has been analysed for a wide range of phytochemicals, vitamins, minerals and dietary fibre components (Shewry *et al*., 2010, 2011; Ward *et al*., 2008; Zhao *et al*., 2009), providing data on genetic diversity and heritability. We have also reported on the analysis of methyl donors such as choline and glycine betaine (Corol *et al*., 2012) by ^1^H‐NMR. This method gives quantitative data on a number of compounds, and in this study, we report on the further application of the high‐throughput ^1^H‐NMR screen to the HEALTHGRAIN wheat material to determine the concentration of asparagine which is the main factor determining the formation of acrylamide in baked cereal products. The data allowed a comprehensive study of the genotype *versus* environmental (GxE) effects on the content of asparagine, which is considered to be the main target for wheat crop improvement towards lower acrylamide potential (Halford *et al*., 2012).

## Results and discussion

### Development of the screen

**Figure 1:**
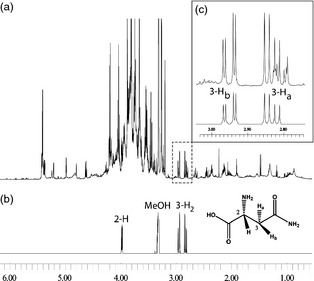
NMR quantitation of asparagine in cereal flours. (a) NMR spectrum of typical wheat flour extract made in CD_3_ OD:D_2_O (1:4). (b) spectrum of pure asparagine made in CD_3_ OD:D_2_O (1:4). (c) expansion of the 3‐H_2_ signal (top) and illustration of 3‐H_b_ of asparagine standard (bottom) which was utilized for quantitation.

^1^H‐NMR profiling of unpurified extracts made directly into deuterated aqueous methanol is a well‐established technique in plant metabolomics (Baker *et al*., 2006; Ward and Beale, 2006; Ward *et al*., 2003). This method is capable of generating metabolite fingerprinting data from large numbers (1000s) of samples. Furthermore, the data are free from alignment and reproducibility problems that are commonly encountered with chromatography‐based analytical techniques, and thus, data from multiple batches of samples collected over several weeks or months can be batch processed with high confidence. Extraction of metabolites involves relatively simple procedures, and there is no need for further derivitisation to ‘view’ metabolites of interest. [^1^H]‐NMR is also absolutely quantitative, irrespective of compound properties, and does not rely on the use of calibration curves. Individual metabolite concentrations can be deduced despite the complex spectra obtained from typical plant or grain samples. Accurate quantitation of individual metabolites in such complex metabolite fingerprints is, however, reliant on the presence of molecule‐specific resonances that are nonoverlapping with other signals in the spectra. A typical NMR spectrum obtained from wheat flour extract is shown in Figure 1, and it can be seen that the characteristic, geminally coupled, signals of the asparagine C‐3 hydrogen atoms fall in a relatively clear area of the spectrum and thus are suitable for integration. Although both of the diastereotopic hydrogens at position C‐3 of asparagine are clearly separated and visible in the NMR spectra of cereal flour extracts, the cleanest signal of the pair at δ2.95 (dd, J = 17 and 4 Hz) arising from 3‐H_b_ was used for integration and quantitation. The signal for 3‐H_a_ at δ2.83 was not utilized due to small interfering signals from aspartate and other metabolites.

After automated alignment and normalization to the d_4_‐TSP internal standard, spectra were reduced to equally sized bins and integration of the selected peaks was accomplished by comparison to the known concentration of the d_4_‐TSP standard. The region taken for asparagine quantitation is δ2.9755–2.9255 representing 3‐H_b_ (1 hydrogen). The high reproducibility of NMR is well documented (Viant *et al*., 2009; Ward *et al*., 2010), and therefore, errors due to instrument drift are minimal. Across three separate extraction replicates, typical relative standard deviations were below 10%.

### Comparison of asparagine contents in wholemeal flour of different wheat species

Integration of binned NMR spectra (circa 10 000 bins of 0.001 ppm each) allowed batch processing of the large number of spectra and accurate quantitation of asparagine. For the lines grown at a single site in 2005, the average contents of asparagine in wholemeal flour samples varied from 0.56 ± 0.14 mg/g d.m. in emmer (*T. turgidum* var. *dicoccum*) (*n *= 5) to 1.12 ± 0.25 mg/g d.m. in einkorn (*T. monococcum* var. *monococcum*) (*n *= 5) (Table 1 and Figure S1). The average contents in winter (*n *= 130) and spring (*n *= 20) bread wheat (*T*. aestivum) genotypes were remarkably similar and were 0.73 ± 0.25 mg/g d.m. and 0.75 ± 0.21 mg/g d.m., respectively, with the contents in the 150 bread wheat lines ranging from 0.32 to 1.56 mg/g d.m. Similarly, the average asparagine content in spelt (*T. aestivum* var. spelta) wholemeal (*n *= 5) was similar to the winter and spring wheat genotypes at 0.72 mg/g d.m, although the range observed in concentration was narrower with variation, in the 5 genotypes studied, of 0.6–0.79 mg/g d.m. Durum wheat (*T. turgidum* var. durum) samples had intermediate levels with an average asparagine concentration of 0.88 mg/g d.m.

**Table 1:** Asparagine Concentrations (mg/g d.m.) in Wholemeal samples from different cereals

Cereal	Number of samples analysed	Average (mg/g d.m.)	Max	Min	CV(%)
Dicoccum	5	0.56 ± 0.14	0.79	0.43	24.00
Durum wheat	10	0.88 ± 0.2	1.32	0.69	22.85
Monococcum	5	1.12 ± 0.25	1.50	0.94	21.88
Spelt	5	0.72 ± 0.08	0.79	0.60	10.85
Spring wheat	20	0.75 ± 0.21	1.40	0.43	27.72
Winter wheat	130	0.73 ± 0.25	1.56	0.32	34.32

### Survey of 150 Bread Wheat Genotypes grown together at a single site

**Figure 2:**
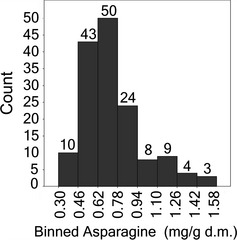
Frequency distribution of 151 bread wheat genotypes based on their asparagine concentration.

Bread wheats make up the greatest proportion of the samples under study. As all lines had been grown at a single location, in the same year, it could be assumed that most of the variation in composition of these samples could be ascribed to the genotype allowing a comparison of asparagine content to be made. The concentration of asparagine in bread wheats typically followed a unimodal distribution which is slightly skewed right (Figure 2). Mean asparagine content of the 150 bread wheats was 0.73 mg/g d.m., whilst the median value was 0.67 mg/g d.m. Fifty (of 150) genotypes contained asparagine contents of between 0.62 and 0.78 mg/g d.m and a further 43 genotypes contained lower concentrations of asparagine, between 0.46 and 0.62 mg/g d.m. A total of 16 genotypes had asparagine levels in excess of 1.1 mg/g d.m., whilst 10 genotypes had very low asparagine contents of between 0.3 and 0.46 mg/g d.m. A full listing of the genotypes grouped into the eight concentration ranges is given in Table S1. The majority of genotypes had asparagine levels between 0.46 and 0.94 mg/g d.m. Those genotypes which did not fall within this range included 3 spring wheat and 31 winter wheat cultivars (Table 2). Of the 10 genotypes with the lowest asparagine content (0.3–0.46 mg/g d.m.), only 1 (Chinese Spring) was spring type, whilst the others (Alba, Bilancia, Blasco, Granbel, Mv‐Emese, Nomade, Palesio, Soissons and Valoris) were winter type. However, it should be noted that only 20 spring wheats were analysed compared with 130 winter wheats. The variation in asparagine content observed here (0.3–1.1 mg/g d.m.) was similar in extent (by over 3.5‐fold) but the values generally higher than those reported for 92 wheat varieties grown in the glasshouse (0.137–0.471 mg/g d.m) (Emebiri, 2014).

**Table 2:** Groupings of bread wheat genotypes showing the highest and lowest concentrations of free asparagine. Data selected from a comparison of 150 bread wheats grown on a single site in the same year (2005)

	Cultivar	Winter (W) or Spring (S) wheat	Asparagine Concentration (mean ± SD)
Low asparagine			
0.32–0.43 mg/g d.m.	Chinese Spring	S	0.43 ± 0.13
	Palesio	W	0.43 ± 0.04
	Blasco	W	0.43 ± 0.03
	Mv‐Emese	W	0.41 ± 0.02
	Bilancia	W	0.40 ± 0.04
	Granbel	W	0.35 ± 0.08
	Soissons	W	0.35 ± 0.04
	Nomade	W	0.33 ± 0.04
	Valoris	W	0.32 ± 0.02
	Alba	W	0.32 ± 0.02
High asparagine			
1.50–1.56 mg/g d.m.	Fleischmann 401	W	1.56 ± 0.06
	Spark	W	1.52 ± 0.07
	Kirkpinar 79	W	1.50 ± 0.11
1.28–1.40 mg/g d.m.	Mexique 50	S	1.40 ± 0.05
	Renan	W	1.37 ± 0.06
	Bankuti 1201	W	1.35 ± 0.07
	Alabasskaja	W	1.28 ± 0.09
1.10–1.25 mg/g d.m	Kirac 66	W	1.25 ± 0.02
	Qualital	W	1.22 ± 0.06
	Blue/A	W	1.16 ± 0.05
	Mv‐Magdalena	W	1.15 ± 0.06
	Tamaro	W	1.15 ± 0.08
	Gerek 79	W	1.15 ± 0.06
	Atlas‐66	W	1.15 ± 0.12
	NS Rana 1	W	1.11 ± 0.11
	Hana	W	1.10 ± 0.04
0.95 1.06 mg/g d.m.	GK‐Tiszataj	W	1.06 ± 0.07
	Karl 92	W	1.06 ± 0.02
	Seu Seun 27	W	1.03 ± 0.08
	Key	W	1.03 ± 0.05
	Probstdorfer Perlo	W	1.02 ± 0.02
	Mv‐Suba	W	0.99 ± 0.03
	Lona	S	0.95 ± 0.04
	Sava	W	0.95 ± 0.06

### Correlation with grain quality

A number of metabolite classes, such as phenolics, sterols and alkylresorcinols, have previously been shown to correlate with grain quality parameters in the HEALTHGRAIN sample set (Rakszegi *et al*., 2008; Shewry *et al*., 2010 and Ward *et al*., 2008). The asparagine concentrations determined in this study were therefore examined against the parameters described in Rakszegi *et al*., 2008; to determine relationships with yield and quality parameters (Table 3). No correlations of asparagine levels were observed between yield, test weight, thousand kernel weight, kernel diameter or mean kernel weight, indicating that final asparagine concentration was not determined by grain size or the total amount of grain produced by the plant. Similarly, the total yields of flour and bran, which will be affected by grain weight, showed no significant relationship with asparagine content. However, a positive correlation (*r *= 0.413, *P *< 0.0001) was observed between asparagine concentration and plant height, with grain from taller plants containing higher final levels of grain asparagine. Breeders have significantly reduced the height of wheats since the second half of the last century, with most modern cultivars being semi‐dwarf. Hence, an inverse correlation was also observed between asparagine concentration and year of cultivar registration with a clear reduction in mean asparagine content being evident from the 1940s onwards (Figure S3).

**Table 3:** Correlations of grain quality parameters with mean asparagine concentration across 150 genotypes grown in Hungary in 2005

Quality parameter	*r*	*P*‐value	Slope
Yield (kg/plot)	0.140	0.0929	Negative
Thousand kernel weight (g/1000 kernel)	0.027	0.7479	Positive
Flour yield (%)	0.138	0.0923	Negative
Chopin bran yield (%)	0.041	0.6225	Positive
Year of registration	**0.255**	**0.0019**	Negative
Plant height (cm)	**0.413**	**<0.0001**	Positive
Mean kernel diameter (mm)	0.129	0.1142	Positive
Mean kernel weight (mg)	0.061	0.4536	Positive
Hardness Index	0.034	0.6753	Positive
Total protein (%)	**0.449**	**<0.0001**	Positive
Wholemeal protein content (%)	**0.507**	**<0.0001**	Positive
Flour protein content flour (%)	**0.376**	**<0.0001**	Positive
Gluten content (%)	**0.437**	**<0.0001**	Positive
Starch content (%)	**0.321**	**<0.0001**	Negative
Moisture content (%)	0.119	0.1443	Positive
Water absorption (%)	**0.354**	**<0.0001**	Positive
Gluten index	0.123	0.133	Positive
Falling number (s)	0.070	0.3916	Positive
Zeleny sedimentation (mL)	**0.366**	**<0.0001**	Positive

Bold values indicate a significant correlation where *p* < 0.05.

Bread‐making quality is determined by a number of parameters, with grain texture and protein content and composition being the most important. In particular, the gluten proteins confer unique rheological properties (visco‐elasticity) to doughs, with strong (highly elastic) doughs being preferred for bread making. We found that the asparagine concentration in whole grain was correlated with total protein per grain (*r *= 0.45, *P* ≤ 0.0001) and also with the % protein in both white flour (*r *= 0.38, *P* ≤ 0.0001) and wholemeal (*r *= 0.51, *P* ≤ 0.0001). Additionally, positive correlations were seen with total gluten (which is itself correlated with grain protein) (*r *= 0.44, *P* ≤ 0.0001) and Zeleny sedimentation (a measure of gluten content and quality) (*r *= 0.37, *P* ≤ 0.0001).

The gluten proteins are poor in asparagine (up to 1 mol% in glutenin subunits, 0.8–2.6 mol% in gliadins) (Shewry *et al*., 2009a), and it is likely that the pool size of free asparagine is regulated at low levels under normal conditions of plant growth. However, asparagine is known to accumulate under conditions of restricted protein synthesis or stress (Lea *et al*., 2007). It is therefore crucial that the developing grain should not be subjected to conditions that may lead to asparagine accumulation, such as restriction of protein synthesis by sulphur limitation, drought or heat stress.

Hard grain texture is required for bread‐making quality as it results in greater starch damage and higher water absorption. Although we observed no correlation between asparagine concentration and grain hardness (measured as hardness index), a positive correlation (*r *= 0.35, *P* ≤ 0.0001) was observed with % water absorption. A weak negative correlation was also observed between asparagine concentration and starch content (*r *= 0.32, *P* ≤ 0.0001). Although some α‐amylase activity is required for bread making, high α‐amylase activity, resulting from prematurity amylase production (PMA) or preharvest sprouting (PHS), adversely affects baking quality. No correlation was observed between falling number, a measure of α‐amylase activity, and asparagine concentration. It is clear, therefore, that asparagine accumulation is positively correlated with factors affecting bread making: protein content and quality and water absorption. It is therefore important for breeders to identify lines which are outside this correlation.

### Correlation with other amino acids and carbohydrates

**Figure 3:**
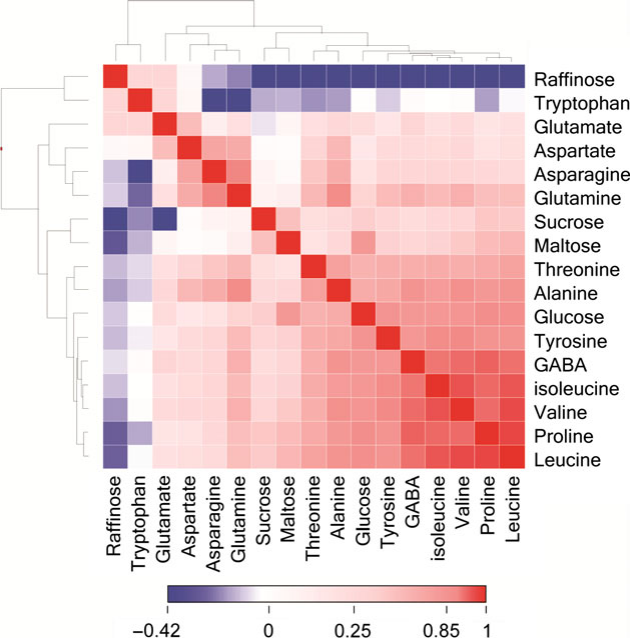
Heatmap of Pearson correlation coefficients of amino acid and soluble carbohydrate levels derived from ^1^H‐NMR metabolite profiling of wholemeal samples of 150 bread wheat genotypes grown in Martonvasar, Hungary in 2005.

^1^H‐NMR has the advantage over targeted methods for amino acid analysis, such as GC‐MS, of being able to analyse compounds from multiple compound classes in the same analysis. In wheat, the major classes of metabolites are soluble carbohydrates and amino acids. Levels of the most abundant of these compounds were therefore examined to determine whether asparagine concentration was correlated with any of the other major co‐extracted components from wheat flour. Figure 3 represents a heatmap of Pearson correlations constructed from the full NMR metabolomics dataset, showing that asparagine concentrations were highly correlated with levels of glutamine, aspartate and alanine. This is perhaps to be expected as asparagine is synthesized from oxaloacetate via aspartate catalysed by a transaminase enzyme. Asparagine synthetase then converts aspartate to asparagine using glutamine, AMP and pyrophosphate. Further but weaker correlations were observed with a number of other amino acids such as GABA, threonine, leucine, valine, isoleucine and proline. There was little correlation with levels of glutamate, and a negative correlation was observed with tryptophan concentration. The relationship of asparagine concentration with soluble sugars was also explored. No correlation was observed between asparagine concentration and the levels of sucrose or maltose, the most abundant sugars in the wheat ^1^H‐NMR spectrum. A weak positive correlation, however, was seen between glucose and asparagine concentrations, whilst the trisaccharide raffinose showed a weak inverse relationship.

### Effect of environmental conditions and growing location on asparagine

Twenty‐six genotypes were selected and grown for two further years in the same location and in three additional locations in the third year. This allowed the effects of environment on asparagine concentration to be explored across diverse growing locations (Table 4).

**Table 4:** Mean asparagine concentrations (mg/g d.m.) of 26 wheat lines grown in six different environments. Values represent the mean (*n *= 3) ± standard deviation

Genotype	Asparagine Concentration (mg/g d.m.)					
	Hungary 2005	Hungary 2006	Hungary 2007	France 2007	UK 2007	Poland 2007
Claire	0.626 ± 0.079	0.542 ± 0.238	1.062 ± 0.075	0.596 ± 0.021	0.493 ± 0.106	0.509 ± 0.009
Lynx	0.728 ± 0.132	0.656 ± 0.141	1.123 ± 0.065	1.061 ± 0.099	0.667 ± 0.071	0.509 ± 0.035
Tommi	0.858 ± 0.014	0.648 ± 0.049	0.740 ± 0.022	0.848 ± 0.065	0.346 ± 0.051	0.523 ± 0.133
CF99105	0.775 ± 0.027	0.601 ± 0.015	0.727 ± 0.067	0.789 ± 0.020	0.477 ± 0.043	0.635 ± 0.042
Obrii	0.575 ± 0.034	0.689 ± 0.079	0.709 ± 0.054	0.669 ± 0.077	0.534 ± 0.039	0.625 ± 0.049
Malacca	0.641 ± 0.073	0.655 ± 0.128	0.958 ± 0.066	0.718 ± 0.072	0.425 ± 0.023	0.477 ± 0.017
Riband	0.664 ± 0.064	0.653 ± 0.033	1.094 ± 0.139	1.021 ± 0.054	0.410 ± 0.036	0.686 ± 0.058
Estica	0.774 ± 0.026	0.570 ± 0.037	0.665 ± 0.038	1.353 ± 0.092	0.336 ± 0.044	0.893 ± 0.029
Rialto	0.694 ± 0.009	0.586 ± 0.049	0.974 ± 0.093	0.627 ± 0.036	0.389 ± 0.069	0.516 ± 0.055
Campari	0.677 ± 0.038	0.777 ± 0.073	0.973 ± 0.059	0.812 ± 0.051	0.439 ± 0.029	0.498 ± 0.015
Avalon	0.649 ± 0.052	0.590 ± 0.032	0.696 ± 0.087	0.765 ± 0.069	0.398 ± 0.087	0.680 ± 0.062
Maris‐Huntsman	0.540 ± 0.089	0.570 ± 0.007	0.586 ± 0.034	0.831 ± 0.062	0.573 ± 0.051	0.468 ± 0.094
Spartanka	0.555 ± 0.023	0.656 ± 0.030	0.707 ± 0.033	0.735 ± 0.064	0.619 ± 0.026	0.627 ± 0.038
Disponent	0.712 ± 0.056	0.747 ± 0.062	0.898 ± 0.052	1.109 ± 0.046	0.436 ± 0.008	0.678 ± 0.062
Atlas‐66	1.146 ± 0.117	0.617 ± 0.075	0.740 ± 0.041	0.519 ± 0.057	0.357 ± 0.019	0.615 ± 0.039
Valoris	0.321 ± 0.024	0.420 ± 0.086	0.501 ± 0.058	0.475 ± 0.025	0.313 ± 0.070	0.419 ± 0.074
Tremie	0.614 ± 0.052	0.727 ± 0.112	0.881 ± 0.071	0.728 ± 0.058	0.531 ± 0.074	0.511 ± 0.026
Gloria	0.472 ± 0.059	0.556 ± 0.011	0.525 ± 0.024	0.599 ± 0.049	0.584 ± 0.112	0.607 ± 0.061
San‐Pastore	0.605 ± 0.052	0.688 ± 0.070	0.718 ± 0.060	1.000 ± 0.022	0.712 ± 0.080	0.800 ± 0.034
Herzog	0.618 ± 0.050	0.661 ± 0.065	0.814 ± 0.041	0.768 ± 0.053	0.463 ± 0.077	0.415 ± 0.037
Mv‐Emese	0.406 ± 0.018	0.415 ± 0.022	0.595 ± 0.073	0.612 ± 0.051	0.621 ± 0.046	0.535 ± 0.059
Isengrain	0.464 ± 0.005	0.569 ± 0.065	0.512 ± 0.078	0.708 ± 0.050	0.386 ± 0.039	0.410 ± 0.039
Crousty	–	0.597 ± 0.014	0.623 ± 0.058	1.132 ± 0.112	0.430 ± 0.034	0.578 ± 0.052
Tiger	–	0.339 ± 0.073	0.598 ± 0.079	0.680 ± 0.060	0.404 ± 0.053	0.522 ± 0.029
Cadenza	0.816 ± 0.034	0.520 ± 0.031	0.695 ± 0.053	0.933 ± 0.065	0.562 ± 0.081	–
Chinese Spring	0.431 ± 0.129	0.939 ± 0.035	0.756 ± 0.052	0.743 ± 0.120	0.428 ± 0.023	–
Mean	0.640 ± 0.171	0.615 ± 0.120	0.764 ± 0.180	0.801 ± 0.208	0.474 ± 0.107	0.572 ± 0.119
CV (%)	26.7	19.6	23.6	26.0	22.6	20.7
Range	0.321–1.146	0.339–0.939	0.501–1.123	0.475–0.801	0.313–0.712	0.410–0.893

When grown at Martonvásár, (Hungary) for 3 years (2005, 2006 and 2007), the concentration of asparagine ranged from 0.32 ± 0.02 mg/g d.m. (cv. Valoris, 2005) to 1.15 ± 0.12 mg/g d.m. (cv Atlas 66, 2005). Values of the means across the 26 genotypes ranged from 0.615 to 0.764 mg/g d.m. and showed a high coefficient of variation (CV = 19.6–26.7%). The variance of individual genotypes due to year of growth ranged from 3.00 to 37.6% (Table 5) with Atlas 66 and Lynx having the highest contents of asparagine (0.834 ± 0.277 and 0.836 ± 0.232 mg/g d.m., respectively) when the data were averaged over the 3 years. It is notable that Atlas 66 is a high protein line which has been used as a source of this trait in breeding programmes (Johnson *et al*., 1985). Similarly, Valoris had the lowest mean concentration (0.414 ± 0.09 mg/g d.m.) over the 3‐year period. The effects of environment were studied further by analysis of the 26 selected lines grown in 2007 in the UK, France and Poland as well as Hungary. Comparison of these samples (grown at 4 locations in a single year) showed a similar pattern in variation in asparagine content (Table 4), from 0.31 mg/g d.m. (Valoris, UK) to 1.12 mg/g d.m. (Lynx, Hungary). The mean values for the 26 lines across the growing locations ranged from 0.47 (UK) to 0.80 (Hungary) mg/g d.m. and showed a high coefficient of variation (CV = 20.7–26.0%). The variance of individual genotypes (Table 5) due to location was typically higher than that observed for the single site comparison over three growing years and ranged from 6.4% (Gloria) to 52.6% (Estica). The contents of asparagine were generally highest in the samples grown in Hungary and France and significantly (*P* < 0.001) lower in those grown in the UK and Poland.

**Table 5:** Statistical comparison of asparagine concentration (mg/g d.m.) of 26 wheat lines grown in 6 environments

	3 Years at a single location (Hungary 2005–2007)			4 Locations in 2007 (Hungary, France, Poland, UK)			6 Environments (Hungary 2005–2007 & France, Poland and UK in 2007)		
	Mean (mg/g d.m.)	Range (mg/g d.m.)	CV (%)	Mean (mg/g d.m.)	Range (mg/g d.m.)	CV (%)	Mean (mg/g d.m.)	Range (mg/g d.m.)	CV (%)
Atlas 66	0.834 ± 0.277	0.617–1.146	33.1	0.558 ± 0.161	0.357–0.740	28.9	0.666 ± 0.267	0.357–1.146	40.1
Avalon	0.645 ± 0.053	0.590–0.696	8.2	0.634 ± 0.162	0.398–0.765	25.6	0.629 ± 0.127	0.398–0.765	20.2
Cadenza	0.677 ± 0.149	0.520–0.816	22.0	0.730 ± 0.188	0.562–0.933	25.7	0.705 ± 0.172	0.520–0.933	24.4
Campari	0.809 ± 0.150	0.677–0.973	18.6	0.680 ± 0.255	0.439–0.973	37.4	0.696 ± 0.201	0.439–0.973	28.9
CF99105	0.701 ± 0.090	0.601–0.775	12.8	0.657 ± 0.135	0.477–0.789	20.6	0.667 ± 0.120	0.477–0.789	17.9
Chinese Spring	0.709 ± 0.257	0.431–0.939	36.3	0.642 ± 0.186	0.428–0.756	28.9	0.659 ± 0.224	0.428–0.939	33.9
Claire	0.743 ± 0.279	0.542–1.062	37.6	0.665 ± 0.269	0.493–1.062	40.4	0.638 ± 0.214	0.493–1.062	33.5
Crousty	0.610 ± 0.018	0.597–0.623	3.0	0.691 ± 0.306	0.430–1.132	44.2	0.672 ± 0.268	0.430–1.132	39.9
Disponent	0.786 ± 0.099	0.712–0.898	12.6	0.780 ± 0.289	0.436–1.109	37.1	0.763 ± 0.226	0.436–1.109	29.6
Estica	0.670 ± 0.102	0.570–0.774	15.3	0.811 ± 0.427	0.336–1.353	52.6	0.765 ± 0.345	0.336–1.353	45.1
Gloria	0.518 ± 0.042	0.472–0.556	8.2	0.579 ± 0.037	0.525–0.607	6.4	0.557 ± 0.051	0.472–0.607	9.2
Herzog	0.698 ± 0.103	0.618–0.814	14.7	0.615 ± 0.205	0.415–0.814	33.3	0.623 ± 0.160	0.415–0.814	25.6
Isengrain	0.515 ± 0.053	0.464–0.570	10.2	0.504 ± 0.146	0.386–0.708	29.0	0.508 ± 0.118	0.386–0.708	23.3
Lynx	0.836 ± 0.252	0.656–1.123	30.1	0.840 ± 0.299	0.509–1.123	35.6	0.791 ± 0.245	0.509–1.123	31.0
Malacca	0.751 ± 0.179	0.641–0.958	23.8	0.644 ± 0.245	0.425–0.958	38.0	0.735 ± 0.231	0.509–1.123	31.4
Maris–Huntsman	0.565 ± 0.023	0.540–0.586	4.1	0.615 ± 0.153	0.468–0.831	24.9	0.595 ± 0.123	0.468–0.831	20.7
MV‐Emese	0.472 ± 0.107	0.406–0.595	22.6	0.591 ± 0.039	0.535–0.621	6.5	0.531 ± 0.098	0.406–0.621	18.4
Obrii	0.658 ± 0.073	0.575–0.709	11.1	0.634 ± 0.075	0.534–0.709	11.8	0.633 ± 0.069	0.534–0.709	10.8
Rialto	0.751 ± 0.201	0.586–0.974	26.7	0.627 ± 0.251	0.389–0.974	40.1	0.631 ± 0.198	0.389–0.974	31.3
Riband	0.803 ± 0.252	0.653–1.094	31.3	0.803 ± 0.316	0.410–1.094	39.4	0.754 ± 0.256	0.410–1.094	34.0
San‐Pastore	0.670 ± 0.059	0.605–0.718	8.8	0.808 ± 0.135	0.712–1.000	16.7	0.754 ± 0.136	0.605–1.000	18.0
Spartanka	0.639 ± 0.078	0.555–0.707	12.1	0.672 ± 0.058	0.619–0.735	8.6	0.650 ± 0.065	0.555–0.735	10.0
Tiger	0.469 ± 0.183	0.339**‐**0.598	39.1	0.551 ± 0.117	0.404–0.680	21.3	0.509 ± 0.139	0.339–0.680	27.3
Tommi	0.749 ± 0.106	0.648–0.858	14.1	0.614 ± 0.224	0.346–0.848	36.5	0.660 ± 0.199	0.346–0.858	30.2
Tremie	0.740 ± 0.134	0.614–0.881	18.1	0.663 ± 0.175	0.511–0.881	26.4	0.665 ± 0.140	0.511–0.881	21.1
Valoris	0.414 ± 0.090	0.321–0.501	21.8	0.427 ± 0.083	0.313–0.501	19.5	0.408 ± 0.077	0.313‐0.501	19.0

**Figure 4:**
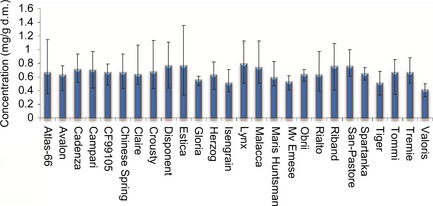
Average asparagine concentrations (mg/g d.m.) over 6 environments (Hungary (2005–2007), France (2007), UK (2007) and Poland (2007). Error bars represent observed asparagine concentration range across the 6 years/locations.

Taking all 6 environments (i.e. multiple sites and years) into account, the genotype with the highest mean asparagine concentration was Lynx (0.79 ± 0.25 mg/g d.m.) (Table 5). Conversely, Valoris contained the lowest asparagine concentration over the 6 environments (0.408 ± 0.08 mg/g d.m.) Despite the large variation due to environment (CV ranged from 9.2% to 45.1%), some genotypes appeared to be more ‘stable’ than others. Figure 4 illustrates the mean asparagine contents across the 6 environmental conditions years for each genotype. Plots are ordered by the observed concentration ranges across the 3 year, multi‐environment study. The most stable lines (exhibiting the lowest concentration range across six conditions) were Gloria, Spartanka and Obrii, whilst those showing the highest variation included Estica, Atlas 66 and Crousty. Thus, despite Valoris having the lowest mean concentration of asparagine across the 6 environments, this genotype is more susceptible to effects of the environment than some of the other genotypes. However, Valoris did show the lowest ‘minimum concentration’ in four of the six environments and thus remains a genotype of interest for lower asparagine content. Likewise, Gloria, Mv‐Emese and Isengrain are potentially interesting to breeders. These, together with Valoris, have significantly lower asparagine levels than other genotypes and under the environments examined in this study, also show the least variation with respect to their growing environment. Conversely, Spartanka and Obrii, which showed low influence of the environment, had higher mean asparagine levels across 6 environments. Thus, when selecting genotypes for low concentrations of asparagine, it is necessary to consider not only the mean concentrations observed but also the range of concentrations observed when grown under different environmental conditions, including trials at multiple locations in different years. This finding is consistent with data reported in Curtis *et al*. (2009) where reports of sevenfold differences were observed in certain genotypes grown across different UK locations in successive years, a range that was larger than that obtained when the same genotypes were grown in the glasshouse.

### Comparison of the ^1^H‐NMR derived data with existing data from GC‐MS

To compare the suitability of ^1^H‐NMR as a method for asparagine quantitation, we compared relevant data in this study to that published on similar lines but analysed using alternate methods such as GC‐MS. Muttucumaru *et al*. (2006) reported asparagine concentrations of 3.07 and 4.43 mmol/kg f.w. for grain derived the cultivar Hereward when grown in field conditions. In our study, using ^1^H‐NMR, we measured an asparagine concentration of 0.59 mg/g dry matter in wholemeal from Hereward plants grown in Hungary in 2005 (Table S1). This equates to 3.90 mmol/kg f.w. and is thus comparable to the Muttucumaru *et al*. study. Grain from the cultivar Malacca has been reported to contain 5.20 mmol/kg f.w. asparagine when grown in a glasshouse (Muttucumaru *et al*., 2006) but significantly less when grown in the field across 6 UK sites in 2006 (0.68–2.79 mmol/kg f.w.) and 2007 (1.65–3.87 mmol/kg f.w.) (Curtis *et al*., 2009). Again, both studies used GC‐MS to determine amino acid concentration. Data from our ^1^H‐NMR study, after conversion to comparable units, returned an asparagine concentration of 2.81 ± 0.15 mmol/kg f.w for grain from this genotype when grown in the UK in 2007. This is therefore within the range of the Curtis *et al*. study. The Curtis *et al*. (2009) study also reported asparagine concentrations for the cultivar Claire of 0.82–2.5 mmol/kg f.w. (across 6 UK sites in 2006) and 2.04–2.68 mmol/kg f.w. (across 6 UK sites in 2007). In our own study, we measured a slightly higher level of asparagine (equivalent to 3.25 mmol/kg f.w.) which although elevated with respect to the Curtis *et al*. (2009) study is still less than the Muttucumaru *et al*. (2006) report of 4.12 mmol/kg asparagine content for this line when grown under glasshouse conditions.

### Effect of environment on the asparagine content of white flours

**Figure 5:**
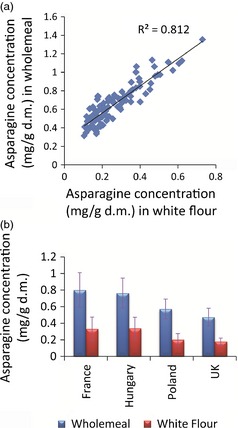
Comparison of asparagine concentration (mg/g d.m.) in white flour and wholemeal samples grown at 4 locations (France, Hungary, UK and Poland) in 2007. (a) correlation of asparagine concentrations (mg/g d.m.) in white flours against wholemeals; (b) mean levels of measured asparagine concentration in white flour and wholemeal samples from each of 4 growing locations. Error bars represent standard deviation of 3 replicate samples.

**Figure 6:**
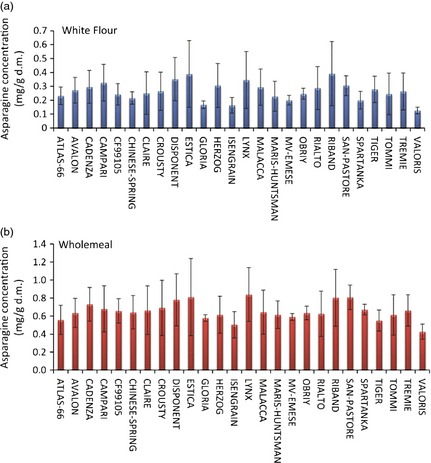
Average asparagine concentrations (mg/g d.m.) from wheat lines grown in 2007 at 4 environments (Hungary, France, UK and Poland). Error bars represent observed asparagine concentration range across the 4 locations. (a) data from white flour samples; (b) data from wholemeal samples.

White flour samples were also analysed for the 26 wheat genotypes grown in 2007 in the 4 locations, and ^1^H‐NMR data of these samples were also collected to determine whether asparagine content located within the starchy endosperm was also subject to similar levels of variation due to environment as that observed in bran‐containing samples of wholemeal. Observed asparagine concentrations (Table S2) in white flour varied from 0.105 ± 0.05 mg/g d.m. (Valoris, 2007, UK) to 0.730 ± 0.06 mg/g d.m. (Estica, 2007, France). The concentration in white flour as a proportion of that in wholemeal varied from 15% (Lynx, 2007, UK) to 57% (Tiger, 2007, France), which agrees with a previous study which showed that asparagine is concentrated in the bran fractions (Shewry *et al*., 2009b). In general, however, the amounts of asparagine present in white flour correlated well (r^2 ^= 0.81) with those observed in the corresponding wholemeal samples (Figure 5a). The effect of environment also followed the pattern observed for wholemeal samples (Figure 5b). Across the 26 lines grown in 4 European locations, the mean asparagine levels were highest in the material grown in France and Hungary (0.33 ± 0.14 and 0.34 ± 0.13 mg/g d.m., respectively) and lowest when the same genotypes were grown in the UK (0.18 ± 0.04 mg/g d.m). The mean levels in material grown in Poland were 0.20 ± 0.07 mg/g d.m. Inspection of the asparagine concentration in white flour for each of the 26 lines, averaged across the 4 locations, compared with the corresponding plots for wholemeal samples, showed that lines which had previously been identified as ‘low asparagine’ in the wholemeal samples also contained the lowest mean level of asparagine when white flour was analysed (Figure 6). Examples included Gloria, Isengrain and Valoris genotypes. The genotypes which showed low environmental variation in the asparagine content of wholemeal (Gloria, MV‐Emese, Obrii, Spartanka, Valoris) also showed the lowest variation in asparagine content in the corresponding white flours. Hence, it should be possible for breeders to select for low asparagine content of white flour by analysis of wholemeal.

### Heritability of asparagine concentration

**Figure 7:**
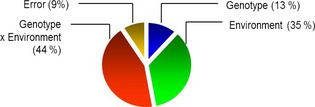
Pie chart showing computed variance components for asparagine concentration in wholemeal samples from 26 bread wheats grown in 6 environments.

The 26 lines described here were grown under a wide range of conditions including different soil types, rainfall and soil water availability, temperature and agricultural practices. Analysis of the grain therefore allowed us to partition the variation between the effects of genotype (G), environment (E), G × E interactions and that which cannot be explained by these factors (termed error). For the asparagine content of wholemeal samples, the ratio of genetic variance to total variance was 0.128, which indicates that only 13% of the observed variation in asparagine content is heritable (Figure 7). By contrast, the variance due to the environment was 36%, whilst that apportioned to genotype x environment was higher at 43%. In fact, the variance due to genotype (13%) was the lowest apart from that assigned to error (9%).

This low ‘broad sense heritability’ is an important observation as it shows that breeders will find it difficult to select for low asparagine content without the availability of specific molecular markers which are not sensitive to environmental factors. Emeberi (2014) reported higher heritability (32%) for asparagine content in wholegrain of 92 wheat varieties. However, these varieties were grown in replicated glasshouse trials in which environmental effects were likely to be much less than in the six highly contrasting environments studied here. The same author also used a genomewide scan to identify 9 single nucleotide polymorphisms (SNPs) which explained between 14% and 24% of the observed variation in asparagine content. This again is indicative of low heritability and suggests that selection for low asparagine will be a challenge for wheat breeders.

### Correlations with Environmental Parameters

Temperature and precipitation data were collected during each growth season (2005–2007) for each of the locations (Figure S2). The mean concentrations of asparagine in wholemeal of the 26 wheat genotypes were used to explore correlations with weather conditions over the growth period at each location (Table 6), with statistically significant correlations being shown in bold. The mean concentration of asparagine showed strong negative correlations (*r *= −0.85, *P* = 0.03) with the total precipitation between heading and harvest dates and also with the total precipitation during the period 3 months before heading date to the harvest date. However, there was no significant correlation with total precipitation during the 3 months before the heading date, indicating that the key time period for effects on asparagine accumulation is during grain development (between the heading and harvest dates). The concentration of asparagine also correlated positively with the mean temperature between heading and harvest dates although the *P*‐value indicated a lower level of significance (*r *= 0.74, *P* = 0.095). As temperatures are generally lower during higher periods of rainfall, it is to be expected that correlations with both temperature and rainfall will be observed. The correlation of asparagine with temperature and inverse correlation with precipitation agree with the data from the 2007 multisite trial where asparagine concentrations were higher in flours from grain grown in Hungary and France (both having higher mean temperatures and lower levels of precipitation) compared to those grown in Poland and the UK, the latter having the lowest mean temperature and the highest amount of rainfall.

**Table 6:** Correlations of temperature, precipitation and soil parameters with mean asparagine concentration across 6 different growing environments

Environmental parameter	*r*	Slope	*P*‐value
Total precipitation from heading date to harvest date (mm)	**0.849**	Negative	**0.033**
Total precipitation from 3 months before heading date to harvest date (mm)	**0.849**	Negative	**0.033**
Mean temperature from heading date to harvest date (°C)	**0.737**	Positive	0.095
Average maximum temp for any 10‐day period between heading and harvest (°C)	**0.541**	Positive	0.268
Average minimum temp for any 10‐day period between heading and harvest (°C)	0.240	Negative	0.646
Total precipitation during 3 months before heading date (mm)	0.074	Positive	0.890

Bold values indicate a significant correlation where *p* < 0.05.

## Conclusions

We have used ^1^H‐NMR profiling to generate quantitative data on asparagine concentrations in large sample sets, using a simple, quick and cheap sample preparation protocol. Analysis by ^1^H‐NMR provided reproducible data with low errors between technical replicates. Comeasurement of a number of different metabolites within the ^1^H‐NMR metabolomics dataset of wholemeal samples of grain allowed levels of asparagine to be compared with other abundant metabolites. We observed a wide variation in asparagine concentration that was highly correlated with certain bread‐making quality parameters such as protein content and water absorption. The asparagine content of white flour was correlated with that of wholemeal with some genotypes having low contents in both fractions. Comparison of 150 bread wheat genotypes, the largest reported study to date, showed that asparagine was typically lower in shorter wheat plants and this was further endorsed by an observed negative relationship between asparagine concentration and the year of registration. This shows that breeding for reduced stature, to maximize yield and harvest index, has also resulted in reduced total grain asparagine.

Comparison of genotypes grown in multiple locations showed a large impact of the environment on asparagine concentration in mature grain with the level of precipitation and temperature during grain development (between heading and harvest) having the greatest effect. Whilst some genotypes could be identified which showed less impact due to the environmental conditions, the overall heritability of asparagine content in whole grain was low (13%), indicating that breeding for low asparagine lines will be more difficult than for many other phenotypic traits. Hence, selection for low asparagine will be a challenge for wheat breeders.

## Materials and methods

### Material

The first field experiment was carried out at Martonvásár (near Budapest, Hungary) in 2004–2005. A total of 150 bread wheat lines were selected to represent the range of diversity in the gene pool available for plant breeders, including wide geographical diversity in origin (from Europe to East Asia, the Americas, and Australia) and including landraces, breeding lines and modern and older cultivars. One hundred and thirty were winter type and 20 were spring type. Five modern cultivars of spelt (a hulled form of hexaploid wheat, *T. aestivum* var. *spelta*), 10 lines of tetraploid durum wheat (*T. turgidum* var. *durum*), five lines each of two early cultivated forms of wheat, diploid einkorn (*T. monococcum* var. *monococcum*) and tetraploid emmer (*T. turgidum* var. *dicoccum*), 10 lines of rye (*Secale cereale*), five lines of oats (*A*v*ena sati*v*a*) and 10 lines of barley (*Hordeum* v*ulgare*) were also included. Full details are given by Ward *et al*. (2008). Twenty‐three of the wheat lines and five rye lines were selected for further studies, together with three additional wheat lines and one additional rye line. These were grown at Martonvásár again in 2005–2006 and 2006–2007 and at Nickerson Seeds UK (Saxham, near Bury St Edmunds, UK), DankoPlant Breeders Ltd. (Choryn, near Poznan, Poland) and the INRA experimental station at Clermont Ferrand (France). Agronomic treatments were standard for the individual sites, with 110 kg of N/Ha being applied in Poland, 204 kg of N/Ha in the United Kingdom, 200 kg of N/Ha in France and 140 kg of N/Ha in Hungary and appropriate use of agrochemicals. Winter, spring and durum wheats were conditioned to 15.5% moisture content before milling, whereas other species were conditioned to 14% moisture content. Milling was carried out using a Perten Laboratory Mill 3100 (with 0.5 mm sieve) and Retsch ZM100 (for *T. monococcum* and oats) to produce wholemeal. Samples were immediately cooled to −20 °C and stored at the same temperature in sealed bags.

## ^1^H‐NMR profiling

NMR sample preparation was carried out according to the procedures described in Ward *et al*. (2003) and Baker *et al*. (2006). NMR extractions into 80:20 D_2_O:CD_3_OD containing 0.05% *d*_4_‐trimethylsilylpropionate (TSP) (1 mL) were performed for three technical replicates, of 30 mg, for each biological sample.

^1^H‐NMR spectra were acquired under automation at 300°K using an Avance Spectrometer (Bruker BioSpin, Coventry, UK) operating at 600.0528 MHz and equipped with a 5 mm selective inverse probe. Spectra were collected using a water suppression pulse sequence with a 90° pulse and a relaxation delay of 5 s. Each spectrum was acquired using 128 scans of 64 000 data points with a spectral width of 7309.99 Hz. Spectra were automatically Fourier transformed using an exponential window with a line broadening value of 0.5 Hz. Phasing and baseline correction were carried out within the instrument software. ^1^H chemical shifts were referenced to d_4_‐TSP at δ0.00.

### Data processing and statistical analysis

^1^H‐NMR spectra were automatically reduced, using Amix (Analysis of MIXtures software; Bruker BioSpin, Rheinstetten, Germany), to ASCII files containing integrated regions or ‘buckets’ of equal width (0.001 ppm for quantitation of asparagine and 0.01 ppm for correlation analysis). Spectral intensities were scaled to the d_4_‐TSP region (δ0.05 to −0.05). The ASCII file was imported into Microsoft Excel for the addition of sampling/treatment details. Regions for individual metabolites were identified via comparison to a library of known standards run under identical conditions. The region used for the quantitation of asparagine was δ2.9755–2.9255. Peaks in this region were integrated against the known concentration of TSP in the sample (0.05% w/v).

Calculations of mean, standard deviations and coefficients of variation were carried out using Microsoft Excel. To relate asparagine concentration values to the physical parameters of the wheat genotypes and to environmental conditions, Pearson correlation coefficients were calculated (from data on a dry weight basis) using Spotfire Decision Site (v. 9.1.2., TIBCO, Somerville, MA).

### G × E analyses

Variance due to genotype and environment was calculated according to methods used in Corol *et al*., 2012. Datasets from the 26 wheat varieties grown in the six different environments were used in statistical models with all effects considered as random to estimate variance components with SAS software (proc VARCOMP). Three technical replicates were used as error terms in the following model: *X* = μ + *E* + *G* + *G* × *E* + ε.

As replicates were technical and not true field replicates, the error term is likely to be an underestimate of the true error. Therefore, we used the ratio σ_*g*_^2^/(σ_*g*_^2^ + σ_*E*_^2^ + σ_*G*×*E*_^2^) as a surrogate to heritability *h*². Indeed, this parameter, although likely to be an underestimate of *h*², is a suitable parameter for plant breeders, as a high value indicates that the trait is mostly affected by the genotype.

## Supporting information


**Figure S1** Mean concentrations (mg/g d.m.) of asparagine in different cereal wholemeal samples grown at a single site in Hungary in 2005.
**Figure S2** Temperature and precipitation data for 6 growing environments used (2005–2007).
**Figure S3** Mean concentrations (mg/g d.m.) of asparagine in winter and spring wheat wholemeal samples grouped by decade of registration.

**Table S1** Mean asparagine concentrations (mg/g d.m.) of winter and spring wheat genotypes grown in Hungary in 2005.

**Table S2** Asparagine concentrations (mg/g d.m.) measured by 1H‐NMR of white flour samples of 26 genotypes grown in 2007 in four European locations.

## References

[pbi12364-bib-0001] Baker, J.M. , Hawkins, N.D. , Ward, J.L. , Lovegrove, A. , Napier, J.A. , Shewry, P.R. and Beale, M.H. (2006) A metabolomic study of substantial equivalence of field‐grown genetically modified wheat. Plant Biotechnol. J. 4, 381–392. 17177804 10.1111/j.1467-7652.2006.00197.x

[pbi12364-bib-0002] Claus, A. , Schreiter, P. , Weber, A. , Graeff, S. , Herrmann, W. , Claupein, W. , Schieber, A. and Carle, R. (2006) Influence of agronomic factors and extraction rate on the acrylamide contents in yeast‐leavened breads. J. Agric. Food Chem. 54, 8968–8976. 17090149 10.1021/jf061936f

[pbi12364-bib-0003] Claus, A. , Carle, R. and Schieber, A. (2008) Acrylamide in cereal products: a review. J. Cereal Sci. 47, 118–133.

[pbi12364-bib-0004] Corol, D.I. , Ravel, C. , Raksegi, M. , Bedo, Z. , Charmet, G. , Beale, M.H. , Shewry, P.R. and Ward, J.L. (2012) Effects of genotype and environment on the contents of betaine, choline and trigonelline in cereal grains. J. Agric. Food Chem. 60, 5471–5481. 22559314 10.1021/jf3008794

[pbi12364-bib-0005] Curtis, T. , Muttucumaru, N. , Shewry, P.R. , Parry, M.A.J. , Powers, S.J. , Elmore, J.S. , Mottram, D.S. , Hook, S. and Halford, N.G. (2009) Effects of genotype and environment on free amino acid levels in wheat grain: implications for acrylamide formation during processing. J. Agric. Food Chem. 57, 1013–1021. 19143525 10.1021/jf8031292

[pbi12364-bib-0006] Curtis, T.Y. , Powers, S.J. , Balagiannis, D. , Elmore, J.S. , Mottram, D.S. , Parry, M.A.J. , Rakszegi, M. , Bedö, Z. , Shewry, P.R. and Halford, N.G. (2010) Free amino acids and sugars in rye grain: implications for acrylamide formation. J. Agric. Food Chem. 58, 1959–1969. 20055414 10.1021/jf903577b

[pbi12364-bib-0007] Emebiri, L. (2014) Genetic variation and possible SNP markers for breeding wheat with low‐grain asparagine, the major precursor for acrylamide formation in heat‐processed products. J. Sci. Food Agric. 94, 1422–1429. 24122675 10.1002/jsfa.6434

[pbi12364-bib-0008] Friedman, M. (2003) Chemistry, biochemistry and safety of acrylamide, a review. J. Agric. Food Chem. 51, 4504–4526. 14705871 10.1021/jf030204+

[pbi12364-bib-0009] Halford, H.G. , Curtis, T.Y. , Muttucumaru, N. , Postles, J. , Elmore, J.S. and Mottram, D.S. (2012) The acrylamide problem: a plant and agronomic science issue. J. Exp. Bot. 63, 2841–2851. 22345642 10.1093/jxb/ers011

[pbi12364-bib-0010] Johnson, V.A. , Mattern, P.J. , Peterson, C.J. and Kuhr, S.L. (1985) Improvement of wheat protein by traditional breeding and genetic techniques. Cereal Chem. 62, 350–355.

[pbi12364-bib-0011] Lea, P.J. , Sodek, L. , Parry, M.A.J. , Shewry, P.R. and Halford, N.G. (2007) Asparagine in plants. Ann. Appl. Biol. 150, 1–26.

[pbi12364-bib-0012] Mottram, D.S. , Wedzicha, B.L. and Dodson, A.T. (2002) Acrylamide is formed in the Maillard reaction. Nature, 419, 448–449. 12368844 10.1038/419448a

[pbi12364-bib-0013] Muttucumaru, N. , Halford, N.G. , Elmore, J.S. , Dodson, A.T. , Parry, M.A.J. , Shewry, P.R. and Mottram, D.S. (2006) The formation of high levels of acrylamide during the processing of flour derived from sulfate‐deprived wheat. J. Agric. Food Chem. 54, 8951–8955. 17090146 10.1021/jf0623081

[pbi12364-bib-0014] Muttucumaru, N. , Elmore, J.S. , Curtis, T. , Mottram, D.S. , Parry, M.A.J. and Halford, N.G. (2008) Reducing acrylamide precursors in raw materials derived from wheat and potato. J. Agric. Food Chem. 56, 6167–6172. 18624429 10.1021/jf800279d

[pbi12364-bib-0015] Poutanen, K. , Shepherd, R. , Shewry, P.R. , Delcour, J.A. , Björck, I. and van der Kamp, J.‐W. (2008) Beyond whole grain: the European HEALTHGRAIN project aims at healthier cereal foods. Cereal Foods World, 53, 32–35.

[pbi12364-bib-0016] Rakszegi, M. , Boros, D. , Kuti, C. , Lang, L. , Bedo, Z. and Shewry, P.R. (2008) Composition and end‐use quality of 150 wheat lines selected for the HEALTHGRAIN diversity screen. J. Agric. Food Chem. 56, 9750–9757. 18921975 10.1021/jf8009359

[pbi12364-bib-0017] Shewry, P.R. , Franklin, J. , Parmar, S. , Smith, S.J. and Miflin, B.J. (1983) The effects of sulphur starvation on the amino acid and protein compositions of barley grain. J. Cereal Sci. 1, 21–31.

[pbi12364-bib-0018] Shewry, P.R. , D'Ovidio, R. , Lafiandra, D. , Jenkins, J.A. , Mills, E.N.C. and Bekes, F. (2009a) Wheat grain proteins Wheat: Chemistry and Technology, 4th edn (Khan K. and Shewry P.R., eds), pp. 223–298. St Paul, MN, USA: AACC.

[pbi12364-bib-0019] Shewry, P.R. , Zhao, F.J. , Gowa, G.B. , Hawkins, N.D. , Ward, J.L. , Beale, M.H. , Halford, N.G. , Parry, M.A. and Abecassis, J. (2009b) Sulphur nutrition differentially affects the distribution of asparagine in wheat grain. J. Cereal Sci. 50, 407–409.

[pbi12364-bib-0020] Shewry, P.R. , Piironen, V. , Lampi, A.‐M. , Edelmann, M. , Kariluoto, S. , Nurmi, T. , Fernandez‐Orozco, R. , Ravel, C. , Charmet, G. , Andersson, A.A.M. , Åman, P. , Boros, D. , Gebruers, K. , Dornez, E. , Courtin, C.M. , Delcour, J.A. , Rakszegi, M. , Bedő, Z. and Ward, J.L. (2010) The HEALTHGRAIN wheat diversity screen: effects of genotype and environment on phytochemicals and dietary fiber components. J. Agric. Food Chem. 58, 9291–9298. 20438061 10.1021/jf100039b

[pbi12364-bib-0021] Shewry, P.R. , Van Schaik, F. , Ravel, C. , Charmet, G. , Rakszegi, M. , Bedo, Z. and Ward, J.L. (2011) Genotype and environment effects on the contents of vitamins B1, B2, B3, and B6 in wheat grain. J. Agric. Food Chem. 59, 10564–10571. 21863876 10.1021/jf202762b

[pbi12364-bib-0022] Stadler, R.H. , Blank, I. , Varga, N. , Robert, F. , Hau, J. , Guy, P.A. , Robert, M.C. and Riediker, S. (2002) Acrylamide from Maillard reaction products. Nature, 419, 449–450. 12368845 10.1038/419449a

[pbi12364-bib-0023] Tareke, E. , Rydberg, P. , Karlsson, P. , Eriksson, S. and Tőrnqvist, M. (2002) analysis of acrylamide, a carcinogen formed in heated foodstuffs. J. Agric. Food Chem. 5, 4998–5006. 12166997 10.1021/jf020302f

[pbi12364-bib-0024] Viant, M.R. , Bearden, D.W. , Bundy, J.G. , Burton, I.W. , Collette, T.W. , Ekman, D.R. , Ezernieks, V. , Karakach, T.K. , Lin, C.Y. , Rochfort, S. , De Ropp, J.S. , Teng, Q. , Tieerdema, R.S. , Walter, J.A. and Wu, H. (2009) International NMR‐based environmental metabolomics intercomparison exercise. Environ. Sci. Tech. 43, 219–225. 10.1021/es802198z 19209610

[pbi12364-bib-0025] Ward, J.L. and Beale, M.H. (2006) NMR spectroscopy in plant metabolomics In Biotechnology in Agriculture and Forestry, Vol 57 Plant Metabolomics (Saito K., Dixon R.A. and Willmitzer L., eds), pp. 81–91. Berlin Heidelberg: Springer‐Verlag.

[pbi12364-bib-0026] Ward, J.L. , Harris, C. , Lewis, J. and Beale, M.H. (2003) Assessment of ^1^H NMR spectroscopy and multivariate analysis as a technique for metabolite fingerprinting of *Arabidopsis thaliana* . Phytochemistry, 62, 949–957. 12590122 10.1016/s0031-9422(02)00705-7

[pbi12364-bib-0027] Ward, J.L. , Poutanen, K. , Gebruers, K. , Piironen, V. , Lampi, A.‐M. , Nyström, L. , Andersson, A.A.M. , Åman, P. , Boros, D. , Rakszegi, M. , Bedő, Z. and Shewry, P.R. (2008) The HEALTHGRAIN cereal diversity screen: concept, results and prospects. J. Agric. Food Chem. 56, 9699–9709. 18921969 10.1021/jf8009574

[pbi12364-bib-0028] Ward, J.L. , Baker, J.M. , Miller, S.J. , Deborde, C. , Maucourt, M. , Biais, B. , Rolin, D. , Moing, A. , Moco, S. , Vervoort, J. , Lommen, A. , Schafer, H. , Humpfer, E. and Beale, M.H. (2010) An inter‐laboratory comparison demonstrates that [H‐1]‐NMR metabolite fingerprinting is a robust technique for collaborative plant metabolomic data collection. Metabolomics, 6, 263–273. 20526352 10.1007/s11306-010-0200-4 PMC2874487

[pbi12364-bib-0029] Zhao, F.J. , Su, Y.H. , Dunham, S.J. , Rakszegi, M. , Bedő, Z. , McGrath, S.P. and Shewry, P.R. (2009) Variation in mineral micronutrient concentrations in grain of wheat lines of diverse origin. J. Cereal Sci. 49, 290–295.

