# Supplementary material for: 1H‐NMR screening for the high‐throughput determination of genotype and environmental effects on the content of asparagine in wheat grain
Source: Plant Biotechnol J. 2015 Mar 27;14(1):128–39. doi: 10.1111/pbi.12364 (PMC4949679; doi:10.1111/pbi.12364)

Figure S1. Mean concentrations (mg/g d.m.) of asparagine in different cereal wholemeal samples grown at a single site in Hungary in 2005. A: error bars represent mean concentration +/- standard deviation; B: Error bars represent the range observed across cultivars. Number of analysed cultivars are indicated at the top of each bar

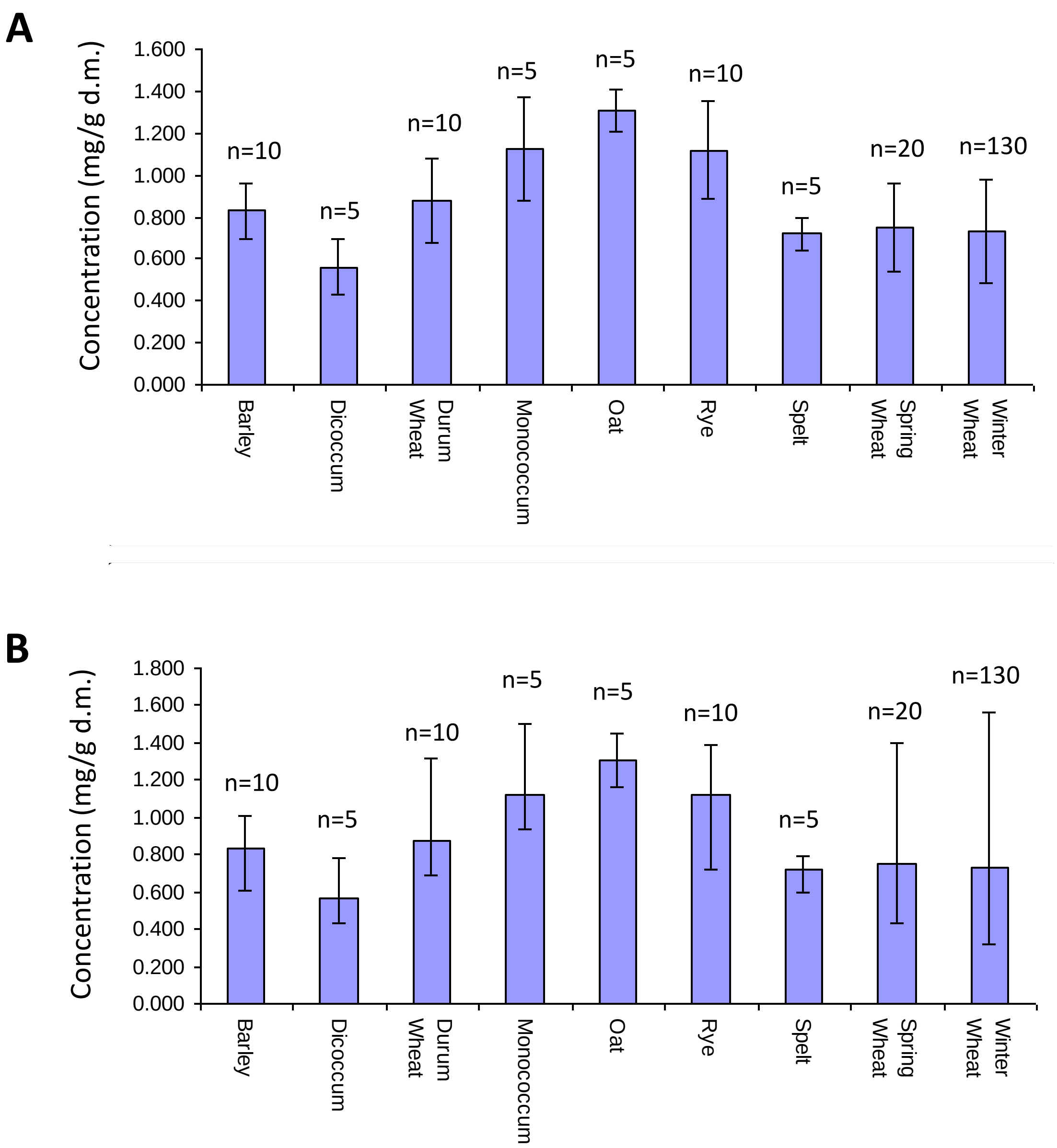

Figure S2: Temperature and precipitation data for 6 growing environments used (2005-2007). A: Temperature; B: Precipitation

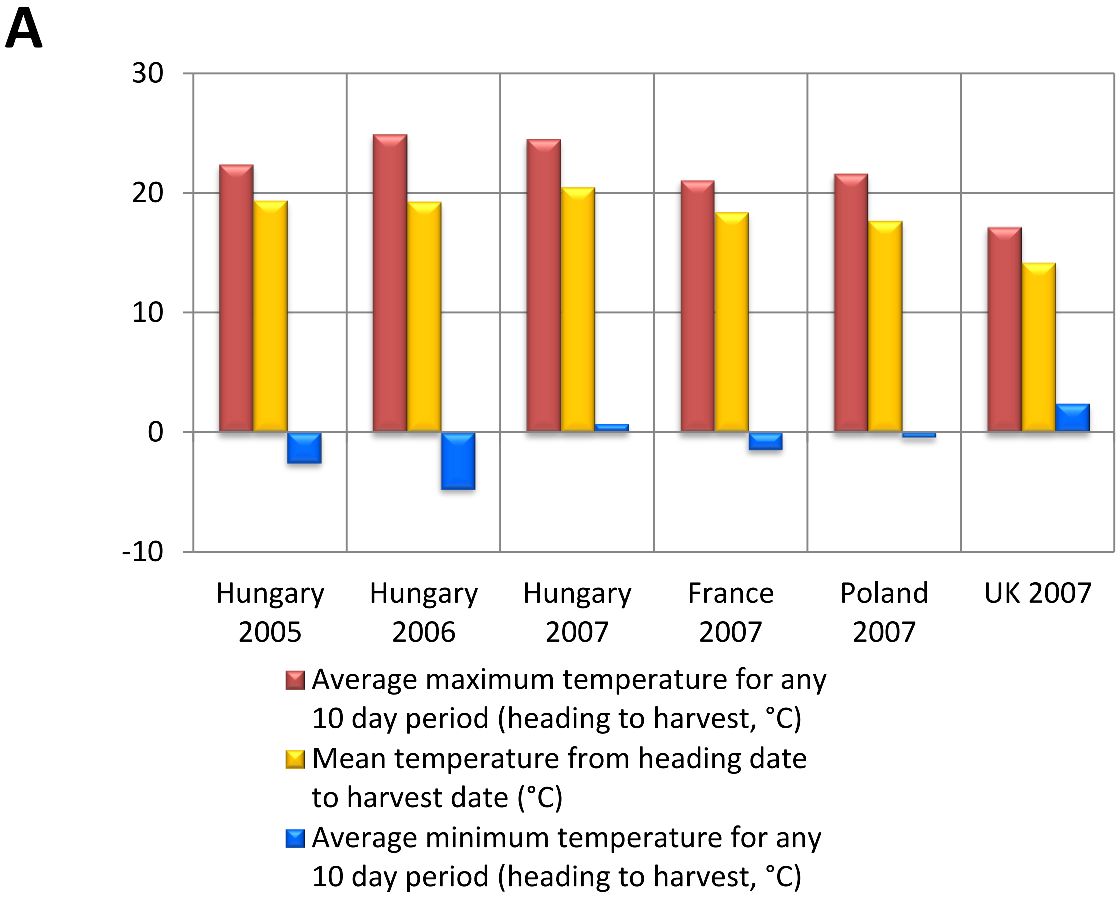

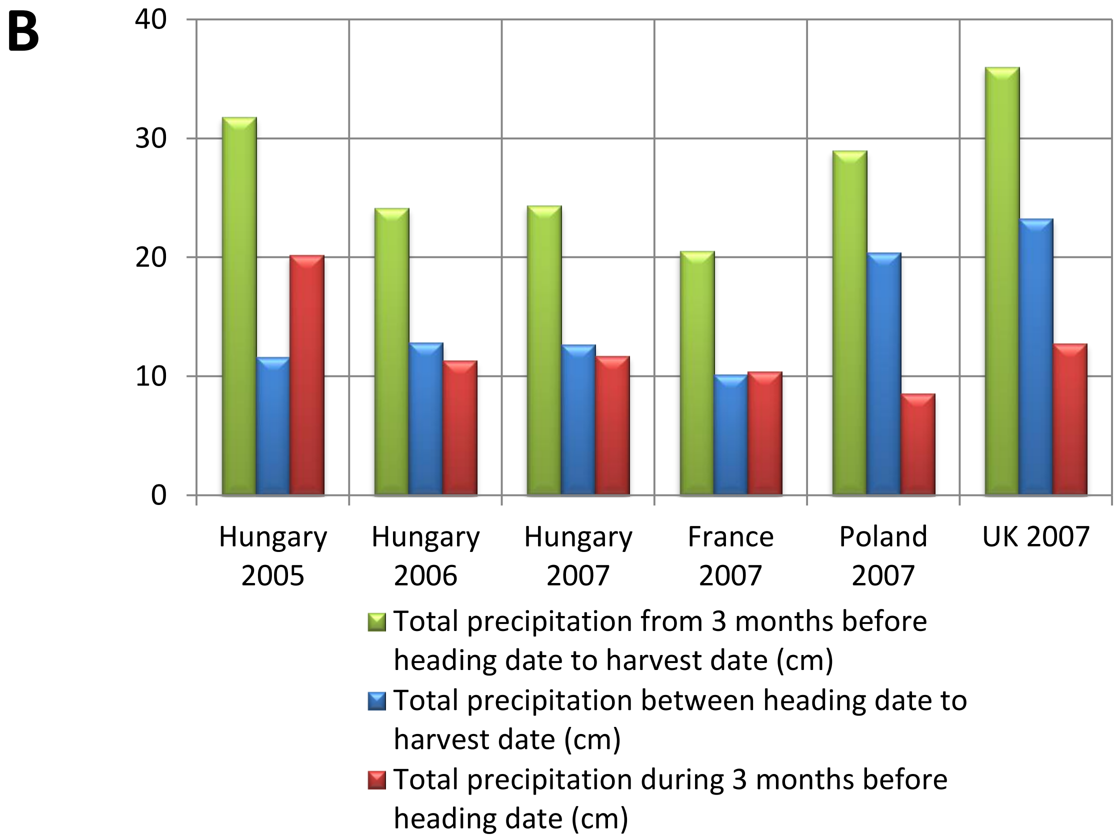

Figure S3. Mean concentrations (mg/g d.m.) of asparagine in winter and spring wheat wholemeal samples grouped by decade of registration. Number of analysed cultivars per decade are indicated at the top of each bar

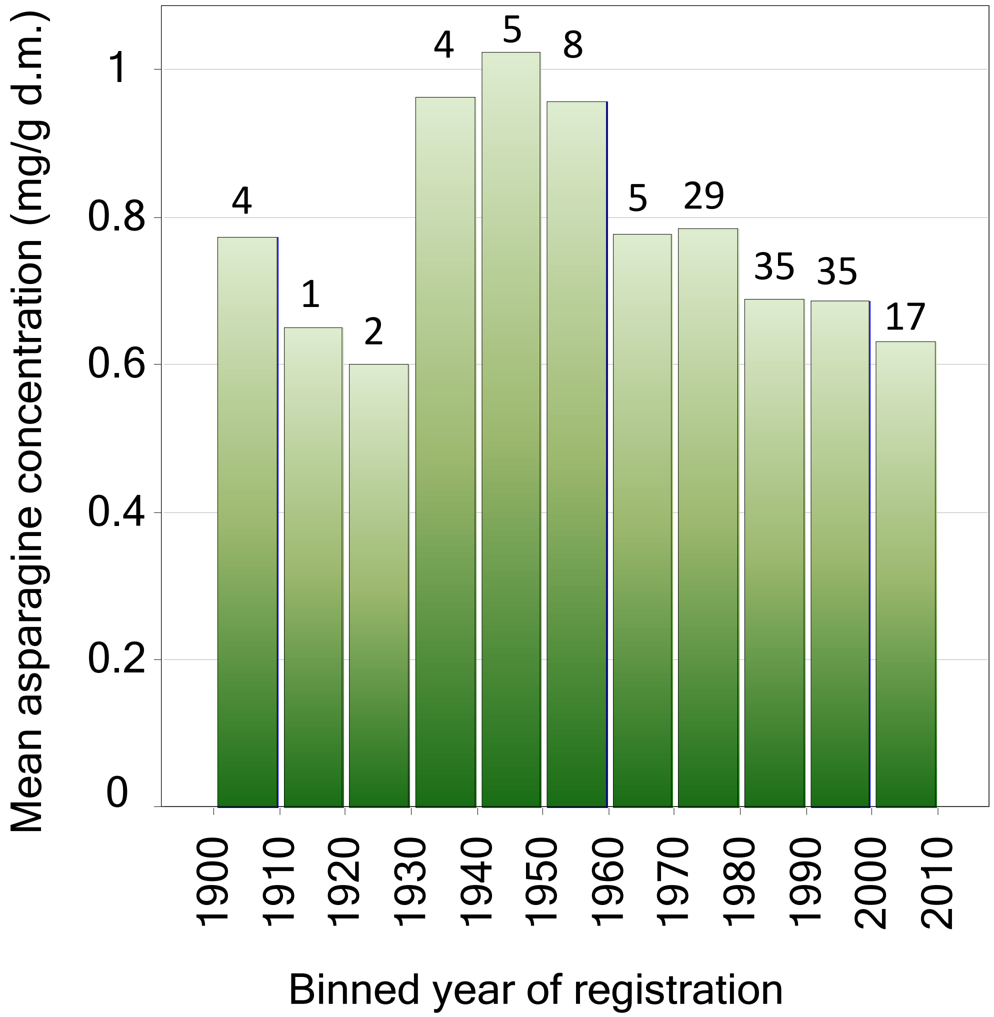

Supplement: Supplementary file 1 — Figure S1 Mean concentrations (mg/g d.m.) of asparagine in different cereal wholemeal samples grown at a single site in Hungary in 2005. Figure S2 Temperature and precipitation data for 6 growing environments used (2005–2007). Figure S3 Mean concentrations (mg/g d.m.) of asparagine in winter and spring wheat wholemeal samples grouped by decade of registration. [file PBI-14-128-s002.pdf]
