# Supplementary material for: 1H‐NMR screening for the high‐throughput determination of genotype and environmental effects on the content of asparagine in wheat grain
Source: Plant Biotechnol J. 2015 Mar 27;14(1):128–39. doi: 10.1111/pbi.12364 (PMC4949679; doi:10.1111/pbi.12364)
Supplement: Supplementary file 2 — Table S1 Mean asparagine concentrations (mg/g d.m.) of winter and spring wheat genotypes grown in Hungary in 2005. [file PBI-14-128-s001.docx]

**Table S1:** Mean asparagine concentrations (mg/g d.m.) of winter and spring wheat genotypes grown in Hungary in 2005. Lines are grouped by concentration range. Values represent the mean of 3 individual replicates.

| **Range**  **(mg/g d.m.)** | **Cultivar** | **Cereal** | **Asparagine (mg/g d.m.)** |
| --- | --- | --- | --- |
|  |  |  |  |
| 1.56 – 1.28 | Fleischmann 481 | Winter wheat | 1.56 ±0.06 |
|  | Spark | Winter wheat | 1.52 ± 0.07 |
|  | Kirkpinar 79 | Winter wheat | 1.50 ± 0.11 |
|  |  |  |  |
| 1.40 – 1.28 | Mexique 50 | Spring wheat | 1.40 ± 0.05 |
|  | Renan | Winter wheat | 1.37 ± 0.06 |
|  | Bankuti 1201 | Winter wheat | 1.35 ± 0.07 |
|  | Alabasskaja | Winter wheat | 1.28 ± 0.09 |
|  |  |  |  |
| 1.25 – 1.10 | Kirac 66 | Winter wheat | 1.25 ± 0.02 |
|  | Qualital | Winter wheat | 1.22 ± 0.06 |
|  | Blue/A | Winter wheat | 1.16 ± 0.05 |
|  | Mv-Magdalena | Winter wheat | 1.15 ± 0.06 |
|  | Tamaro | Winter wheat | 1.15 ± 0.08 |
|  | Gerek 79 | Winter wheat | 1.15 ± 0.06 |
|  | Atlas-66 | Winter wheat | 1.15 ± 0.12 |
|  | NS Rana 1 | Winter wheat | 1.11 ± 0.11 |
|  | Hana | Winter wheat | 1.10 ± 0.04 |
|  |  |  |  |
| 1.06 – 0.95 | GK-Tiszataj | Winter wheat | 1.06 ± 0.07 |
|  | Karl 92 | Winter wheat | 1.06 ± 0.02 |
|  | Seu Seun 27 | Winter wheat | 1.03 ± 0.08 |
|  | Key | Winter wheat | 1.03 ± 0.05 |
|  | Probstdorferperlo | Winter wheat | 1.02 ± 0.02 |
|  | Mv-Suba | Winter wheat | 0.99 ± 0.03 |
|  | Lona | Spring wheat | 0.95 ± 0.04 |
|  | Sava | Winter wheat | 0.95 ± 0.06 |
|  |  |  |  |
| 0.93 – 0.79 | Ble des Domes | Winter wheat | 0.93 ± 0.02 |
|  | Chara | Spring wheat | 0.91 ± 0.09 |
|  | Red Fife | Spring wheat | 0.91 ± 0.04 |
|  | Krasnodarskaya 99 | Winter wheat | 0.90 ± 0.06 |
|  | Fertodi 293 | Winter wheat | 0.89 ± 0.04 |
|  | Nap Hal | Winter wheat | 0.89 ± 0.05 |
|  | Frederick | Winter wheat | 0.89 ± 0.03 |
|  | Kotuku | Winter wheat | 0.86 ± 0.09 |
|  | Tommi | Winter wheat | 0.86 ± 0.01 |
|  | Azteca 67 | Spring wheat | 0.86 ± 0.04 |
|  | Su321 | Winter wheat | 0.85 ± 0.04 |
|  | Milan | Spring wheat | 0.85 ± 0.06 |
|  | Balkan | Winter wheat | 0.84 ± 0.05 |
|  | Yumai 34 | Winter wheat | 0.83 ± 0.02 |
|  | Stephens | Winter wheat | 0.83 ± 0.03 |
|  | Augusta | Winter wheat | 0.83 ± 0.08 |
|  | Atay 85 | Winter wheat | 0.82 ± 0.01 |
|  | Cadenza | Spring wheat | 0.82 ± 0.03 |
|  | Cardinal | Winter wheat | 0.80 ± 0.04 |
|  | Libellula | Winter wheat | 0.79 ± 0.05 |
|  | Klein Estrella | Winter wheat | 0.79 ± 0.09 |
|  | Autonomia | Winter wheat | 0.79 ± 0.07 |
|  | Kukri | Spring wheat | 0.79 ± 0.12 |
|  |  |  |  |
| 0.78 – 0.63 | Capo | Winter wheat | 0.78 ± 0.05 |
|  | Sumai 3 | Winter wheat | 0.78 ± 0.05 |
|  | CF99105 | Winter wheat | 0.78 ± 0.03 |
|  | Estica | Winter wheat | 0.77 ± 0.03 |
|  | Manital | Winter wheat | 0.77 ± 0.06 |
|  | Caphorn | Winter wheat | 0.76 ± 0.04 |
|  | Monopol | Winter wheat | 0.75 ± 0.02 |
|  | Pastor | Spring wheat | 0.75 ± 0.15 |
|  | Scout 66 | Winter wheat | 0.73 ± 0.05 |
|  | Camp Remy | Winter wheat | 0.73 ± 0.04 |
|  | Biscay | Winter wheat | 0.73 ± 0.03 |
|  | Lynx | Winter wheat | 0.73 ± 0.13 |
|  | Carmen | Winter wheat | 0.73 ± 0.03 |
|  | Amadeus | Winter wheat | 0.72 ± 0.06 |
|  | Cubus | Winter wheat | 0.72 ± 0.05 |
|  | Iljicovka | Winter wheat | 0.72 ± 0.08 |
|  | CF99007 | Winter wheat | 0.71 ± 0.04 |
|  | Disponent | Winter wheat | 0.71 ± 0.06 |
|  | Sunstar | Spring wheat | 0.70 ± 0.06 |
|  | Rialto | Winter wheat | 0.69 ± 0.01 |
|  | CF99075 | Winter wheat | 0.69 ± 0.04 |
|  | Ornicar | Winter wheat | 0.69 ± 0.03 |
|  | Plainsman V | Winter wheat | 0.68 ± 0.03 |
|  | Kanzler | Winter wheat | 0.68 ± 0.03 |
|  | Etoile de Choisy | Winter wheat | 0.68 ± 0.01 |
|  | Campari | Winter wheat | 0.68 ± 0.04 |
|  | Alliance | Winter wheat | 0.68 ± 0.05 |
|  | Courtot | Winter wheat | 0.67 ± 0.07 |
|  | Geronimo | Winter wheat | 0.67 ± 0.04 |
|  | Aurora | Winter wheat | 0.67 ± 0.04 |
|  | Thatcher | Spring wheat | 0.67 ± 0.04 |
|  | B16 | Winter wheat | 0.67 ± 0.04 |
|  | Glenlea | Spring wheat | 0.67 ± 0.17 |
|  | Riband | Winter wheat | 0.66 ± 0.06 |
|  | Apache | Winter wheat | 0.66 ± 0.03 |
|  | Agron | Winter wheat | 0.66 ± 0.02 |
|  | Manitoba | Spring wheat | 0.66 ± 0.03 |
|  | Bezostaya | Winter wheat | 0.65 ± 0.03 |
|  | Avalon | Winter wheat | 0.65 ± 0.05 |
|  | Tam 200 | Winter wheat | 0.65 ±0.06 |
|  | Produttore | Winter wheat | 0.64 ±0.04 |
|  | Akteur | Winter wheat | 0.64 ±0.03 |
|  | Malacca | Winter wheat | 0.64 ±0.07 |
|  | Roussalka | Winter wheat | 0.64 ±0.07 |
|  | Janz | Spring wheat | 0.64 ±0.03 |
|  | Flamura | Winter wheat | 0.63 ±0.01 |
|  | Zvezda | Winter wheat | 0.63 ±0.09 |
|  | Claire | Winter wheat | 0.63 ±0.08 |
|  | Recital | Winter wheat | 0.63 ±0.08 |
|  |  |  |  |
| 0.62 – 0.46 | Galahad | Winter wheat | 0.62 ±0.02 |
|  | Tremie | Winter wheat | 0.61 ±0.05 |
|  | Arthur 71 | Winter wheat | 0.61 ±0.06 |
|  | Vona | Winter wheat | 0.61 ±0.01 |
|  | Momtchil | Winter wheat | 0.61 ±0.03 |
|  | San-Pastore | Winter wheat | 0.60 ±0.05 |
|  | CF99102 | Winter wheat | 0.60 ±0.04 |
|  | Millenium | Winter wheat | 0.60 ±0.17 |
|  | Lasta | Winter wheat | 0.60 ±0.06 |
|  | Yubileinaya 50 | Winter wheat | 0.59 ±0.08 |
|  | Gene | Winter wheat | 0.59 ±0.06 |
|  | Mvpalotas | Winter wheat | 0.59 ±0.06 |
|  | Hereward | Winter wheat | 0.59 ±0.04 |
|  | Dekan | Winter wheat | 0.59 ±0.04 |
|  | Sultan 95 | Spring wheat | 0.59 ±0.02 |
|  | Sadovo-1 | Winter wheat | 0.58 ±0.02 |
|  | Obrii | Winter wheat | 0.57 ±0.03 |
|  | Baranjka | Winter wheat | 0.57 ±0.04 |
|  | Fundulea | Winter wheat | 0.57 ±0.07 |
|  | Pan | Spring wheat | 0.56 ±0.11 |
|  | Spartanka | Winter wheat | 0.55 ±0.02 |
|  | Buck Catriel | Winter wheat | 0.55 ±0.05 |
|  | Red River 68 | Spring wheat | 0.55 ±0.05 |
|  | Catbird | Spring wheat | 0.55 ±0.03 |
|  | Ellvis | Winter wheat | 0.55 ±0.03 |
|  | Martonvasari 17 | Winter wheat | 0.55 ±0.04 |
|  | Pobeda | Winter wheat | 0.55 ±0.03 |
|  | Ukrainka | Winter wheat | 0.54 ±0.07 |
|  | Maris-Huntsman | Winter wheat | 0.54 ±0.09 |
|  | Skorospelka 3B | Winter wheat | 0.54 ±0.05 |
|  | Albatrosodeskii | Winter wheat | 0.54 ±0.03 |
|  | Korweta | Winter wheat | 0.53 ±0.04 |
|  | Moulin | Winter wheat | 0.53 ±0.04 |
|  | Sagittorio | Winter wheat | 0.51 ±0.01 |
|  | Guarni | Winter wheat | 0.51 ±0.07 |
|  | Taldor | Winter wheat | 0.51 ±0.08 |
|  | Ravenna | Winter wheat | 0.51 ±0.09 |
|  | Mieti | Winter wheat | 0.48 ±0.08 |
|  | Arina | Winter wheat | 0.48 ±0.06 |
|  | Thesee | Winter wheat | 0.48 ±0.06 |
|  | Gloria | Winter wheat | 0.47 ±0.06 |
|  | Isengrain | Winter wheat | 0.46 ±0.01 |
|  | Begra | Winter wheat | 0.46 ±0.02 |
|  |  |  |  |
| 0.43 – 0.32 | Chinese-Spring | Spring wheat | 0.43 ±0.13 |
|  | Palesio | Winter wheat | 0.43 ±0.04 |
|  | Blasco | Winter wheat | 0.43 ±0.03 |
|  | Mv-Emese | Winter wheat | 0.41 ±0.02 |
|  | Bilancia | Winter wheat | 0.40 ±0.04 |
|  | Granbel | Winter wheat | 0.35 ±0.08 |
|  | Soissons | Winter wheat | 0.35 ±0.04 |
|  | Nomade | Winter wheat | 0.33 ±0.04 |
|  | Valoris | Winter wheat | 0.32 ±0.02 |
|  | Alba | Winter wheat | 0.32 ± 0.02 |
|  |  |  |  |
