# Supplementary material for: 1H‐NMR screening for the high‐throughput determination of genotype and environmental effects on the content of asparagine in wheat grain
Source: Plant Biotechnol J. 2015 Mar 27;14(1):128–39. doi: 10.1111/pbi.12364 (PMC4949679; doi:10.1111/pbi.12364)
Supplement: Supplementary file 3 — Table S2 Asparagine concentrations (mg/g d.m.) measured by 1H‐NMR of white flour samples of 26 genotypes grown in 2007 in four European locations. [file PBI-14-128-s003.docx]

**Table S2.** Asparagine concentrations (mg/g d.m.) measured by ^1^H-NMR of white flour samples of 26 genotypes grown in 2007 in four European locations. Values in brackets represent the ratio of asparagine in white flour to that in corresponding wholemeal samples.

|  | Asparagine concentration mg/g d.m (ratio of asparagine in white flour to wholemeal) | | | |
| --- | --- | --- | --- | --- |
| Genotype | Hungary | France | Poland | UK |
| Campari | 0.494 ± 0.097 (0.49) | 0.361 ± 0.005 (0.44) | 0.256 ± 0.008 (0.50) | 0.192 ± 0.042 (0.28) |
| Herzog | 0.453 ± 0.050 (0.53) | 0.430 ± 0.070 (0.54) | 0.192 ± 0.002 (0.45) | 0.147 ± 0.056 (0.31) |
| Disponent | 0.399 ± 0.072 (0.42) | 0.549 ± 0.039 (0.48) | 0.261 ± 0.093 (0.37) | 0.200 ± 0.073 (0.45) |
| Tommi | 0.350 ± 0.005 (0.46) | 0.399 ± 0.012 (0.46) | 0.118 ± 0.031 (0.22) | 0.114 ± 0.035 (0.32) |
| Tremie | 0.457 ± 0.052 (0.50) | 0.237 ± 0.039 (0.32) | 0.154 ± 0.017 (0.29) | 0.206 ± 0.027 (0.37) |
| CF99105 | 0.313 ± 0.006 (0.41) | 0.305 ± 0.043 (0.37) | 0.188 ± 0.070 (0.29) | 0.164 ± 0.028 (0.33) |
| Valoris | 0.160 ± 0.088 (0.31) | 0.123 ± 0.022 (0.25) | 0.114 ± 0.011 (0.27) | 0.105 ± 0.047 (0.32) |
| Isengrain | 0.179 ± 0.020 (0.34) | 0.237 ± 0.028 (0.32) | 0.108 ± 0.022 (0.25) | 0.129 ± 0.052 (0.32) |
| Claire | 0.476 ± 0.049 (0.43) | 0.211 ± 0.025 (0.34) | 0.135 ± 0.041 (0.26) | 0.181 ± 0.047 (0.36) |
| Maris-Huntsman | 0.204 ± 0.026 (0.33) | 0.379 ± 0.014 (0.44) | 0.121 ± 0.014 (0.25) | 0.209 ± 0.022 (0.46) |
| Lynx | 0.622 ± 0.038 (0.53) | 0.381 ± 0.021 (0.35) | 0.172 ± 0.039 (0.32) | 0.207 ± 0.022 (0.15) |
| Malacca | 0.480 ± 0.038 (0.48) | 0.285 ± 0.083 (0.38) | 0.223 ± 0.058 (0.41) | 0.187 ± 0.091 (0.42) |
| Rialto | 0.506 ± 0.004 (0.50) | 0.282 ± 0.039 (0.44) | 0.200 ± 0.054 (0.37) | 0.162 ± 0.026 (0.40) |
| Riband | 0.606 ± 0.052 (0.53) | 0.573 ± 0.075 (0.54) | 0.232 ± 0.031 (0.33) | 0.155 ± 0.021 (0.36) |
| Avalon | 0.247 ± 0.045 (0.34) | 0.339 ± 0.067 (0.43) | 0.352 ± 0.090 (0.51) | 0.152 ± 0.021 (0.37) |
| San-Pastore | 0.258 ± 0.028 (0.35) | 0.379 ± 0.030 (0.36) | 0.353 ± 0.094 (0.38) | 0.231 ± 0.032 (0.31) |
| Estica | 0.302 ± 0.012 (0.44) | 0.730 ± 0.056 (0.53) | 0.354 ± 0.073 (0.38) | 0.170 ± 0.018 (0.49) |
| Gloria | 0.198 ± 0.050 (0.37) | 0.158 ± 0.025 (0.25) | 0.134 ± 0.025 (0.21) | 0.180 ± 0.026 (0.30) |
| Spartanka | 0.175 ± 0.030 (0.24) | 0.178 ± 0.051 (0.23) | 0.151 ± 0.017 (0.23) | 0.294 ± 0.078 (0.46) |
| Obrii | 0.295 ± 0.018 (0.40) | 0.260 ± 0.088 (0.38) | 0.208 ± 0.034 (0.32) | 0.222 ± 0.025 (0.40) |
| Atlas-66 | 0.320 ± 0.030 (0.42) | 0.227 ± 0.066 (0.42) | 0.211 ± 0.044 (0.33) | 0.170 ± 0.040 (0.44) |
| Crousty | 0.231 ± 0.036 (0.36) | 0.464 ± 0.023 (0.40) | 0.218 ± 0.048 (0.36) | 0.146 ± 0.033 (0.33) |
| Tiger | 0.298 ± 0.024 (0.48) | 0.401 ± 0.073 (0.57) | 0.236 ± 0.038 (0.44) | 0.178 ± 0.060 (0.42) |
| Mv-Emese | 0.211 ± 0.054 (0.34) | 0.209 ± 0.076 (0.33) | 0.149 ± 0.036 (0.27) | 0.233 ± 0.085 (0.36) |
| Chinese-Spring | 0.265 ± 0.017 (0.34) | 0.177 ± 0.061 (0.23) | - | 0.205 ± 0.054 (0.35) |
| Cadenza | 0.326 ± 0.043 (0.46) | 0.395 ± 0.047 (0.41) | - | 0.166 ± 0.015 (0.28) |
|  |  |  |  |  |
